# Dysregulation of Lipid and Glucose Metabolism in Nonalcoholic Fatty Liver Disease

**DOI:** 10.3390/nu15102323

**Published:** 2023-05-16

**Authors:** Neha Bhat, Arya Mani

**Affiliations:** Cardiovascular Research Center, Department of Internal Medicine, Yale School of Medicine, New Haven, CT 06511, USA

**Keywords:** lipid metabolism, substrate flux, insulin resistance, type II diabetes, NAFLD

## Abstract

Non-Alcoholic Fatty Liver Disease (NAFLD) is a highly prevalent condition affecting approximately a quarter of the global population. It is associated with increased morbidity, mortality, economic burden, and healthcare costs. The disease is characterized by the accumulation of lipids in the liver, known as steatosis, which can progress to more severe stages such as steatohepatitis, fibrosis, cirrhosis, and even hepatocellular carcinoma (HCC). This review focuses on the mechanisms that contribute to the development of diet-induced steatosis in an insulin-resistant liver. Specifically, it discusses the existing literature on carbon flux through glycolysis, ketogenesis, TCA (Tricarboxylic Acid Cycle), and fatty acid synthesis pathways in NAFLD, as well as the altered canonical insulin signaling and genetic predispositions that lead to the accumulation of diet-induced hepatic fat. Finally, the review discusses the current therapeutic efforts that aim to ameliorate various pathologies associated with NAFLD.

## 1. Introduction

Non-Alcoholic Fatty Liver Disease (NAFLD) currently affects over 25% of the adult population globally [1,2]. If left untreated, the fatty liver can progress to more severe stages such as steatohepatitis (NASH), fibrosis, cirrhosis, and hepatocellular carcinoma, and an increased risk of developing type II diabetes and cardiovascular diseases. This is primarily due to the malfunctioning of the liver affecting flux through glucose and lipid metabolic pathways, thereby affecting the regulation of lipid and glucose levels in the body.

Recent studies have shown that patients with NAFLD have a greater than two-fold risk of developing type II diabetes [3], and those with type II diabetes have a 75% prevalence of NAFLD [1,2,4]. Both NAFLD and type II diabetes are associated with an increased risk of cardiovascular diseases [5]. These findings suggest the need for increased surveillance of NAFLD in patients with cardiovascular diseases and type II diabetes, and vice versa. Therapies developed for treating type II diabetes or cardiovascular diseases may also help reduce cardiovascular complications in patients with NAFLD, and vice versa where patients with NAFLD could benefit from therapies against type II diabetes. Moreover, NAFLD has also become one of the most common liver diseases in the pediatric population, with a 5–10% prevalence globally [6,7,8,9]. These patients are also susceptible to developing cardiovascular diseases and type II diabetes, making it important to carry out prospective follow-ups to prevent serious outcomes. Hence, there is an urgent need to develop novel therapies and investigate their efficacies in ongoing and prospective clinical trials.

### 1.1. Altered Carbon Flux in NAFLD

The liver plays a crucial role in regulating lipid homeostasis. After digestion, triglycerides (TGs) are transported in lipoprotein particles called chylomicrons. These TGs are broken down by lipoprotein lipase in the capillaries, releasing fatty acids that are taken up by adipose and skeletal muscle tissues. The remaining chylomicron remnants are then taken up by the liver. The liver also synthesizes lipids from dietary sugars through de novo lipogenesis (DNL). Hepatic TGs are packaged and secreted along with free and esterified cholesterol as very low-density lipoprotein particles (VLDL). Through lipolysis of triglycerides and exchange of apolipoproteins, VLDL transforms into intermediate-density lipoprotein particles (IDL) and low-density lipoprotein particles (LDL) [10]. Furthermore, the liver receives free fatty acids through lipolysis of TGs in the adipose tissue, particularly during fasting or insulin resistance states [11]. In this review, we examine how carbon from lipids and carbohydrates flows through various metabolic pathways in the liver and how this flux becomes disrupted in cases of over-nutrition. This is a complex condition since energy flux through multiple pathways is intertwined with signaling from endocrine hormones such as insulin, glucagon, adipokines, cytokines, and myokines.

The pathological response to an excess of carbon flux from energy-rich nutrients, such as sugars and lipids, suggests that metabolic pathways are vulnerable to managing excess nutrients. This vulnerability largely arises due to systemic insulin resistance, which is one of the first predictors of dysregulated lipid and glucose metabolism [1,2,12,13,14,15,16]. Insulin resistance, along with visceral adiposity, elevated triglycerides, and reduced high-density lipoproteins (HDL), are common clinical characteristics of NASH patients, including women with gestational diabetes [1,2,12,13,14,15,16].

**Figure 1:**
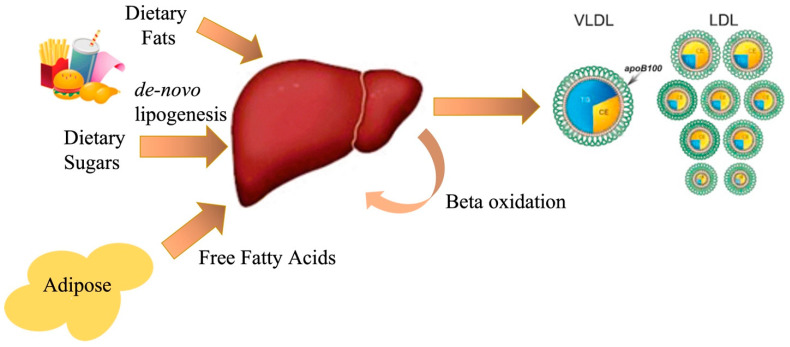
The flow of lipids from the intestine to the adipose tissue and liver, and from the liver into systemic circulation.

Insulin resistance leads to dysregulation of both anabolic (de novo synthesis and lipid accumulation) and catabolic (oxidation and secretion) processes of lipid and glucose metabolism (Figure 1). One of the immediate effects of insulin resistance is impaired glucose disposal in peripheral skeletal muscle [17,18,19]. Normally, insulin binding and phosphorylation of insulin receptors activate a downstream cascade of reactions that culminate in the translocation of glucose transporter Glut4 to the plasma membrane, facilitating glucose uptake by the skeletal muscle [17,18,19,20]. In an insulin-resistant state, the Glut4 receptor fails to translocate to the membrane in the skeletal muscles, hindering the uptake of plasma glucose. As a consequence, the glycogen stores of the muscle are depleted, which is one of the first manifestations of insulin resistance [21,22,23]. The lack of energy stores in the skeletal muscle leads to wasting and sarcopenia in type II diabetic patients [24]. Persistently higher levels of plasma glucose cause increased insulin secretion from the beta cells of the pancreas [13,19,25,26,27,28,29]. The resulting hyperinsulinemia is one of the defining features of dysregulated glucose metabolism and NAFLD. Continued hyperinsulinemia appears to desensitize insulin signaling in skeletal muscle, hepatocytes, and adipocytes, further exacerbating systemic insulin resistance [17,18,19]. The mechanisms causing this desensitization are only beginning to be understood (see below, structure of insulin receptor) [11,30,31,32,33,34].

**Figure 2:**
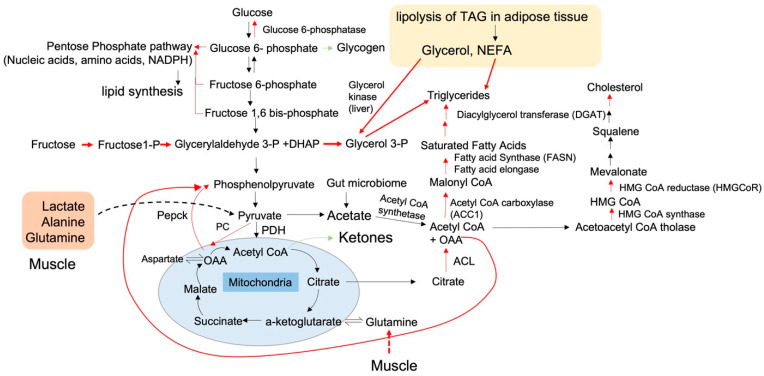
A review of carbon flux through glycolysis, the Krebs cycle, and lipid, amino, and nucleic acid synthesis pathways in NAFLD. The glucose enters the hepatocytes through Glut2 transporters independently of insulin and undergoes phosphorylation by hexokinases, trapping glucose intracellularly. Glucose 6-phosphate can be metabolized in three ways: by being stored as glycogen typically after a meal, by being metabolized into nucleic acids, amino acids, and NADPH through the pentose phosphate pathway, or by being broken down into glyceraldehyde 3-phosphate (GAP) and DHAP (di-hydroxy acetone phosphate) in glycolysis. Other contributors to the GAP pool are fructose and glycerol, the latter being released as a byproduct of lipolysis from adipose tissue. Fructose is readily taken up by the liver and broken down by fructose 1-P aldolase into GAP. GAP is a major contributor to the pyruvate pool and serves as a backbone for triglycerides. The pyruvate pool receives major contributions from lactate, alanine, and glutamine released by the muscle. The pyruvate pool, in equilibrium with lactate, can be converted to acetate or contribute to acetyl coA or oxaloacetate (OAA). Acetyl CoA contributes to ketogenesis and acetate production or is cycled through the Kreb’s cycle. The acetyl-CoA combines with OAA to produce citrate, which can be shuttled out of mitochondria and reconverted to acetyl CoA and OAA. Acetyl CoA can generate ketones, especially during fasting, and/or serve as a precursor for triglycerides and cholesterol synthesis. The OAA is exported out through a malate shuttle and is converted to phosphoenol pyruvate (PEP) through the action of the gluconeogenic enzyme PEPCK (phosphor enol pyruvate carboxy kinase). Carbon flux through the various anabolic (lipid, glucose, nucleic, and protein synthesis) and catabolic (glycolysis, beta-oxidation, and Krebs–Electron transport chain) pathways is influenced by the overall nutritional load and the status of insulin sensitivity. Insulin sensitivity regulates the flux through lipid synthesis and glucose-producing pathways in the hepatocytes. The pathways highlighted in red are increased in NAFLD.

Under normal conditions, insulin suppresses lipolysis of TGs from the adipose tissue and suppresses glucose production from the liver. Insulin resistance causes increased lipolysis of TGs from adipose tissue and increased glucose production in the liver. The increased lipolysis of TGs in the adipose tissue increases plasma levels of non-esterified fatty acids (NEFA) and glycerol [11,30,31,32,33,34]. Upon entry into hepatocytes, NEFA is esterified into TGs by the action of Glycerol 3-phosphate acyltransferase3 (GPAT) and Diacylglycerol O-transferase1 (DGAT). The other product of adipose tissue lipolysis is Glycerol, which is converted into glycerol-3-phosphate (G3P) in the liver by Glycerol-3 kinase (G3K). The increased flux of glycerol to the liver increases gluconeogenic flux, leading to hyperglycemia [32,35,36] (Figure 2). In NAFLD, G3P is increasingly diverted toward the glycolysis/TCA cycle [37] and gets oxidized to generate oxaloacetate (OAA), which is then converted to phosphoenolpyruvate (PEP) by mitochondrial phosphoenolpyruvate carboxykinase (PEPCK), the rate-limiting enzyme in the gluconeogenesis pathway. This results in increased glucose production from the liver (Figure 2). Moreover, the reductive equivalents generated in the TCA cycle and their subsequent oxidation lead to endoplasmic reticulum (ER) stress [31,38] and reactive oxygen species production, resulting in steatohepatitis [39]. In addition to providing carbons for gluconeogenesis, G3P also serves as a carbon backbone for the esterification of acyl chains by the actions of GPAT and DGAT, thereby contributing to lipid synthesis [37,40].

Despite resistance to insulin action in adipose tissue, de-novo lipogenesis (DNL), an insulin-dependent process, is stimulated during insulin resistance in the liver. During DNL, excess sugars are diverted towards fatty acid synthesis pathways in hepatocytes as demonstrated by stable isotope studies [11]. While glucose and fructose-rich diets stimulate DNL in the liver [11,41,42], labeled tracers of these sugars do not proportionally convert to fatty acids, indicating that they do not directly convert to fatty acids [43]. The gluconeogenic precursors such as alanine, lactate, and glutamine were found to be direct contributors to carbon in fatty acid synthesis through the DNL pathway [44,45]. Furthermore, gut microbiota-derived acetate has recently been shown to increase DNL in response to dietary fructose [46]. The acetyl-CoA generated by lipogenic substrates is released from the mitochondria as citrate, which is converted to acetyl-CoA and oxaloacetate (OAA) in the cytoplasm by citrate lyase [47]. The cytoplasmic acetyl-CoA is directed toward fatty acid synthesis via the DNL pathway, while OAA is converted to phosphoenolpyruvate (PEP) by cytoplasmic PEPCK for gluconeogenesis. The fatty acids generated by the DNL pathway (Figure 2) are esterified by glycerol released from adipose tissue. Therefore, the pathways of gluconeogenesis and lipogenesis are intricately linked and are simultaneously fueled by carbons from glycerol and NEFA released by adipose tissue, acetate from gut microbiota, and circulating acetate, lactate, and alanine.

The contribution of altered beta-oxidation towards the development of NAFLD is likely context-dependent. While some studies report decreased mitochondrial oxidation of fatty acids in NAFLD [48], others indicate a compensatory increase in beta-oxidation [31,38,39,41,49]. A recent report suggested the presence of increased flux through the TCA cycle and reduced ketogenesis without any change in beta-oxidation per se in patients with NAFLD [49]. These discrepancies are likely due to differences in underlying disease mechanisms and/or adaptive mechanisms that counter disease states [48,50]. In conclusion, the altered flux of glycerol, NEFA, and fatty acids increases lipid synthesis and glucose production in the liver, leading to a vicious cycle of hyperlipidemia and hyperglycemia and fatty liver disease.

### 1.2. What Condition Comes First: Insulin Resistance or NAFLD?

The question of whether insulin resistance or NAFLD comes first has remained unanswered. Individuals with metabolic disorders, manifested as NAFLD, type II diabetes, and metabolic syndrome, display a range of heterogenous traits: visceral adiposity, hyperlipidemia, hypertension, atherogenic dyslipidemia, glucose intolerance, prediabetes, and insulin resistance. Several investigators have made attempts to simplify this heterogeneity by classifying patients into different phenotypic classes [51,52,53]. This entails that the disease is highly heterogenous, there is no single cure for all the metabolic defects, and hence, therapeutic strategies have to become increasingly personalized. One factor that can advance precision medicine is a comprehensive knowledge of the underlying genetic background of the disease. While genome-wide association studies have linked many genetic loci to various metabolic traits, common variants that underlie the association of these traits have remained vastly unidentified [51]. Apart from genetic background, the composition of consumed nutrients by individuals can influence how metabolic dysfunction manifests. A diet rich in high fructose corn syrup, used as an additive to processed foods, has been shown to stimulate lipogenesis in the liver [46,54,55]. Similarly, a diet disproportionally enriched in saturated fats may increase the atherogenic lipid particles in the plasma. It is likely that there is not a single linear relationship between the development of insulin resistance and hepatic steatosis, and the mechanistic studies detailed below further show this.

The mechanistic studies in rodents show that hepatic lipid accumulation suppresses insulin signaling and, conversely, insulin resistance stimulates hepatic lipid accumulation. This mutually stimulating cycle causes both hyperglycemia and hyperlipidemia, culminating in T2D and NAFLD. In support of hepatic lipid accumulation suppressing insulin signaling, investigators have shown that lipid accumulation in the liver increases the pool of sn-1,2-diacylglycerol (DAG) in the membrane, which recruits protein kinase C (PKC) epsilon to the membrane. PKCepsilon then suppresses insulin-induced phosphorylation of insulin receptor beta at Y1162 and promotes inhibitory phosphorylation of threonine at residue 1160, suggesting that lipid accumulation inhibits insulin signaling [34,56,57,58,59]. As discussed previously, insulin resistance increases hepatic lipid accumulation by reducing peripheral lipid disposal, increasing adipose tissue lipolysis, and stimulating DNL in the liver [1,11,15,18,19,20,27,41,42,60,61,62,63]. Another example showing the complex relationship between hepatic lipogenesis and insulin resistance is illustrated by insulin-mediated activation of the mammalian target of rapamycin-1 (mTORC1). The activation of canonical insulin signaling results in a cascade of substrate phosphorylations, which inhibits Tumor sclerosis complex 1 and 2 (TSC1/2). TSC1/2 inhibition leads to the activation of mTORC1 through the G-protein Rheb complex [64]. Previously, mTORC1 activation was believed to be necessary for lipogenesis [65,66]. However, soon it was realized that the prolonged activation of mTORC1 negatively feedbacks and dampens insulin signaling [67] by its downstream targets such as S6K1 (Ribosomal Protein S6 Kinase beta 1). S6K1 promotes the inhibitory serine phosphorylation of insulin receptor substrate 1 (IRS1) [68,69]. Therefore, mTORC1 promotes lipogenesis in the liver in response to insulin, but then inhibits insulin signaling upon prolonged activation.

The autonomous insulin signaling activation of hepatocytes is further augmented by the resident macrophages in the liver known as Kupffer cells, which greatly influence overall glucose and lipid homeostasis [70,71]. From rodent studies, it is evident that Kupffer cells are activated into a pro-inflammatory state when fed a high-fat diet. These activated macrophages secrete chemokines that recruit monocytes from the plasma called monocyte-derived macrophages (MoMF) [72,73] and together secrete pro-inflammatory cytokines such as TNFalpha, IL-6, IL1-beta, which suppresses insulin signaling in the hepatocytes [70,71,74,75,76,77] and activate hepatic stellate cells leading to fibrosis [78]. Conversely, other studies suggest that Kupffer cells modulate hepatic steatosis by secreting anti-inflammatory cytokines that stimulate insulin signaling in the hepatocytes [74,79,80,81]. Taken together, these studies indicate a regulatory function of Kupffer cells on hepatic insulin sensitivity and hepatic steatosis. One consistent observation in these different studies is that a pro-inflammatory milieu worsens the phenotype of hepatic steatosis and hence, the diets that reduce inflammation should be recommended for patients suffering from metabolic dysfunction. Altogether, lipid accumulation and insulin resistance mutually stimulate each other in the liver through autonomous and non-autonomous pathways and cascade into progressively worsening conditions such as steatohepatitis and type II diabetes. The key question is: “what is the leading condition in any given individual”. Accordingly, the focus should be on the identification of unique biomarkers that can classify patients based on these conditions and treating them in a personalized fashion.

### 1.3. The Intersection of Metabolic Flux with the Activities and Transcript Levels of Metabolic Enzymes

The metabolic flux through different pathways is tightly regulated by the expression and/or activity of key rate-limiting enzymes. These rate-limiting enzymes control the flux through different pathways. The substrate flux in each pathway varies depending on the metabolic needs (energy generation or energy conservation) and the metabolic state (insulin-sensitive or insulin-resistant). In a metabolic pathway, there are critical flux-altering nodes that determine whether a cell will be engaged in anabolic or catabolic processes. An example of such a flux-altering node is when the cell decides whether to engage the substrate pyruvate in a decarboxylation reaction towards acetyl-CoA by pyruvate dehydrogenase and promote glucose oxidation or towards carboxylation by pyruvate carboxylase to generate OAA. The latter is then converted to the gluconeogenic precursor PEP (Figure 2). Likewise, acetyl-CoA can be directed towards oxidation in the TCA cycle or exported out of the mitochondria as citrate for lipid synthesis depending upon the ATP levels (Figure 2). Another example of a flux-altering critical node is whether acetyl-CoA is diverted towards ketogenesis, which is a catabolic reaction, or towards cholesterol synthesis, which is anabolic. Depending on the metabolic state, the HMG-CoA generated from acetyl-CoA, can be diverted towards ketogenesis by 3-hydroxy-3-methylglutaryl-CoA lyase (HMG-CoA lyase) or towards cholesterol biosynthesis by 3-hydroxy-3-methylglutaryl-CoA reductase (HMGCR). In sum, whether a given substrate is used for an anabolic or catabolic reaction is determined by the expression levels of key metabolic enzymes and the metabolic state of the cells such as ATP content, nutrient availability, and insulin sensitivity. In NAFLD, the flux is directed toward anabolic pathways. For instance, pyruvate is directed toward gluconeogenesis [32] and acetyl-CoA is directed toward lipid [11] and cholesterol synthesis [11,49].

The endocrine hormones insulin and glucagon exert a potent influence in determining the choice between anabolic versus catabolic pathways. Insulin transcriptionally and post-translationally activates sterol regulatory binding protein 1c (Srebp1-c), which upregulates hepatic lipogenesis [82,83,84,85,86,87,88,89]. Insulin activates the mammalian target of rapamycin complex2 (mTORC2), protein kinase B (PKB), and liver X receptor (LXRa), which transcriptionally activates the expression of Srebp1c [89,90,91]. The genetic mouse models reveal that Srebp-1c, ChRebp1a, LXRa, and mTORC2, increase the expression of enzymes in the glycolytic pathway concomitant with increasing the enzyme levels of the DNL pathway [89,90,91]. Increased levels of glycolytic intermediates such as glucose 6-phosphate, fructose, and the pentose phosphate pathway intermediate xylulose-5-phosphate activate the transcription factors carbohydrate response element binding protein 1a (ChRebp1a) and ChRebp1b [85,90,92,93,94]. ChRebp1a and ChRebp1b are phosphorylated and translocate to the nucleus where they transcriptionally activate genes that promote lipogenesis. The increased glycolysis likely diverts the carbon flux towards an increased lipogenesis [95,96]. However, detailed in vivo flux studies using tracers are essential to confirm this hypothesis and how these processes are disrupted in insulin-resistant conditions. This is especially important in light of observations that DNL, an insulin-dependent process, remains insulin sensitive despite systemic insulin resistance [63]. In contrast to insulin signaling, glucagon–PKA signaling inactivates Srebp-1c by inducing inhibitory phosphorylation [97,98], as well as blocking the nuclear translocation of ChRebp1 by the glucagon-cAMP-PKA signaling pathway [94,99,100].

Previous studies from our group revealed that impaired Wnt signaling in a mouse model of human LRP6^R611C^ mutation results in the activation of the mTORC1-SREBP1c axis and the development of NAFLD [101,102]. The genetic gain and loss of TCF4 (Transcription Factor 4), an effector of Wnt signaling, further confirmed the protective action of Wnt signaling against NAFLD [103]. TCF4 transcriptionally activates Fgf19 in the intestinal epithelium, which suppresses bile synthesis in the liver, prevents dietary lipid uptake from the intestine, and protects against diet-induced fatty liver [103]. Accordingly, an independent study by Novartis reported that the loss of hepatic Wnt/β-catenin activity by Lgr4/5 deletion led to impaired secretion of bile acids, cholestasis, and altered lipid homeostasis, leading to the development of NAFLD [104]. Our group recently discovered another mutation, R102C, in dual specificity tyrosine phosphorylation regulated kinase 1b (Dyrk1b), that is strongly linked with metabolic syndrome in the carriers [105]. Further mechanistic studies revealed that Dyrk1b spontaneously causes fatty liver in rodents by increasing de novo lipogenesis and hepatic fatty acid uptake [56,105,106,107,108], despite suppressing the [104] canonical insulin signaling by PKC epsilon-mediated insulin receptor inactivation [57,59]. Our model recapitulates the selective insulin resistance model previously suggested by other investigators [63]. The detailed mechanistic studies revealed that Dyrk1b increases the activity of the mammalian target of rapamycin complex2 (mTORC2), a stimulator of DNL in the liver [91], in a kinase-independent manner and stimulates the autophosphorylation of mTOR. Importantly, the Dyrk1b mRNA and protein levels were elevated in mouse liver with diet-induced NAFLD and in human NASH samples. Since Dyrk1b is activated by auto-phosphorylation during its translation, transcriptional and post-transcriptional regulation seems to be the most probable mechanism to regulate its activity in the cells and [109,110,111] future studies are required to clarify these findings.

### 1.4. Selective Insulin Resistance in Hepatocytes: A Perspective from the Structure of Insulin Receptors

The concept of selective insulin resistance was proposed by Brown and Goldstein about 15 years ago when they made a striking observation that systemic insulin resistance impairs the insulin-dependent lowering of plasma glucose, but the insulin-dependent lipogenesis in the liver remains increased [63]. One contributor to NAFLD is the unrestrained supply of fatty acids from the lipolysis of adipose tissue in an insulin-resistant state [33]. While this may explain the 60% contribution to hepatic fat in NAFLD patients, the increase of fatty acid synthesis by the DNL pathway still remains unexplained [11,112]. Insulin stimulates DNL by transcriptional activation of key lipogenic enzymes such as sterol regulatory binding protein 1c (Srebp1c), carbohydrate response element binding protein (ChRebp1), and upstream transcription factor 1 (USF1), and by post-transcriptional regulation of key lipogenic enzymes such as Srebp1c [83]. A potential explanation for this “pathogenic paradox” may be offered by the structural changes in the insulin receptor in response to hyperinsulinemia, which is commonly associated with metabolic syndrome [13]. In a normal unstimulated state, the heterodimeric insulin receptor assumes a symmetrical inverted “V”-shape where the N-terminal end of one alpha subunit interacts with the juxta membrane domains on the other alpha subunit (See references [113,114,115,116] for details). The insulin receptor can bind maximally four insulin molecules [113,114,117,118,119] but the binding of even one insulin molecule to the high-affinity site1 on the insulin receptor is sufficient for the activation of the receptor and its dramatic conformational change from an inverted “V” to an asymmetrical “T” structure [113,114,117,118,119], which is sufficient for downstream signal transduction. These morphological alterations lead to autophosphorylation activation of the receptor, although the precise mechanisms by which insulin induces these conformational changes in the insulin receptor are unclear [114]. It has been observed that a fully occupied (all four insulin binding sites) insulin receptor causes the distance between trans-activation domains to increase as opposed to an asymmetrical partially occupied receptor [113]. It is plausible that a fully occupied receptor, as might be present in hyperinsulinemia, is competent to stimulate the lipogenic pathway but is unable to suppress glycogenolysis and gluconeogenesis. Hyperinsulinemia may desensitize the insulin receptor to suppress glycogenolysis and gluconeogenesis but not DNL. Alternatively, hyperinsulinemia [20,63,120,121,122] may differentially activate insulin receptor substrate-1 (IRS-1) versus IRS-2 in NAFLD [123,124]. Further, more clarity is needed to define the structural changes in the IR in an insulin-resistant state and to determine if activation of the insulin receptor is necessary for lipogenesis in NAFLD after the onset of fatty liver disease. An alternative explanation, independent of canonical insulin signaling, could be that transcriptomic and proteomic changes induced in the liver by the nutritional overflow may increase the expression of factors that stimulate lipogenesis even in the absence of active insulin signaling. One such factor that was recently discovered is Dyrk1b [105,108], which is increased transcriptionally in fatty liver disease. Dyrk1b then activates the central regulator of lipogenesis, mTORC2 [91], in the absence of the canonical insulin signaling and in a manner independent of Dyrk1b’s kinase activity. Dyrk1b increases the flux and expression of enzymes in the DNL pathway [56] while canonical insulin signaling is inhibited.

### 1.5. A Discrepancy in the Association of Insulin Resistance with Hepatic Fat Content

A strong positive correlation exists between liver triglyceride content and hepatic insulin resistance in the diet-induced models of NAFLD in rodents and humans [2,13,31,63,120,125,126]. As mentioned previously, increased levels of diacylglycerol (DAG), a precursor to triacylglycerol, correlates with insulin resistance, as DAG has been shown to increase PKCepsilon translocation to the plasma membrane, which causes inhibitory phosphorylation in the beta chain of the insulin receptor [34,56,57,59,127]. However, in several rodent genetic models and human genetic models of NAFLD, the association between hepatic fat and insulin resistance does not hold true. The genetic rodent models such as hepatic Akt1/2 and the hepatic knockout of mTORC2 function show reduced lipogenesis but increased insulin resistance [91,122,128,129]. Downstream of Akt, mTORC1 activation promotes lipogenesis in the liver, as revealed by pharmacological inhibition with rapamycin [66,121]. One caveat to the studies examining the function of rapamycin is that prolonged rapamycin treatment inhibits both mTORC1 and mTORC2 [130]. Notably, the loss of mTORC2 has a potent effect on the prevention of hepatic steatosis and increasing hepatic glucose production [91]. The regulation of hepatic lipogenesis by mTORC1 is more complicated. Both the activation of mTORC1 by disruption of TSC1 and loss of Raptor, a mTORC1 specific subunit, protect against NAFLD–NASH [25,131,132,133], suggesting complex regulation of hepatic lipogenesis by mTORC1. This can be explained by a negative feedback mechanism by which the overactivated mTORC1 turns off its own activation [67]. To delineate mechanisms that selectively activate lipogenesis and avoid activation of negative feedback pathways by mTOR complex1, a recent study by Gosis et al. found that mTORC1 activates lipogenesis by phosphorylating transcription factor E3/B (TFE3/B) which then suppresses fatty-acid oxidation and lysosomal and mitochondrial biogenesis [134]. These findings, however, do not recapitulate the diet-induced models of metabolic syndrome in which lipogenesis and glucose production are both elevated. Another condition in which hepatic steatosis exhibits no correlation with diet-induced insulin resistance is the increased hepatic triglyceride content in homozygous patatin-like phospholipase domain containing 3 (PNPLA3) pI148M mutation carriers, which do not develop hepatic insulin resistance [135,136,137,138]. These studies indicate that despite the common phenotype of hepatic lipid accumulation, the heterogeneity of the underlying mechanisms results in different metabolic states. In-depth analyses of dysregulated signaling pathways in NAFLD are necessary to identify novel drug targets and pursue the management of patients with NAFLD in a personalized fashion [63,120,121].

### 1.6. Therapeutic Interventions to Treat NAFLD

NAFLD begins with a benign fatty acid accumulation but can progress into a pathologically and morphologically complex disease, often associated with CVD and type II diabetes. Therapeutic interventions for preventing type II diabetes are also being investigated for their impacts on alleviating steatosis in the liver (Table 1). These include insulin-sensitizing PPAR agonists, satiety-promoting GLP-1 agonists, and inhibition of the glucose-absorbing Sglt2 cotransporter in the renal tubules. However, as steatosis progresses into inflammation, fibrosis, and HCC, it does not remain merely a metabolic disease. Therefore, other therapies have to be developed to cure advanced disease states such as inflammation, fibrosis, and hepatocellular carcinoma. To that end, Cenicriviroc (CVC), an oral dual CCR2/CCR5 antagonist was studied in the Centaur trial, a randomized, double-blind, placebo-controlled, multinational study of 289 adults with histological evidence of NASH and liver fibrosis. Although the drug failed to demonstrate a statistically significant improvement in the primary endpoint of NASH improvement, defined as ≥2-point improvement in NAS, it showed improvement in fibrosis, especially in subjects with higher disease activity [139]. Nevertheless, the most successful clinical trials have been limited to those that target primary metabolic defects (Table 1). The FDA has yet to approve a therapy for NAFLD.

## Author Contributions

N.B. and A.M. wrote the manuscript. All authors have read and agreed to the published version of the manuscript.

## Figures and Tables

**Table 1:** Therapeutics for NAFLD and their mechanism of action.

	Mechanism of Action	T2D	Steatosis	Fibrosis	Other Indications	References
PPAR agonist	Insulin sensitizing, transcriptional control	yes	Yes; trials ongoing	Yes; trials ongoing	Weight gain Bone fractures	[140,141,142]
GLP-1 agonist	Increases insulin secretion, enhances satiety	yes	Yes; trials ongoing	Not tested	Weight loss	[143,144,145]
Thyroid receptor beta-agonist	Increases mitochondrial respiration and breakdown of lipids.	Not tested	Yes; trials ongoing	Yes; trials ongoing	Liver and isoform-specific	[146,147,148]
Fgf21	Increases energy expenditure; improves glucose and lipid balance	Not tested	Yes; trials ongoing	Yes; trials ongoing	Some Fgf21 analogues were discontinued	[149,150,151]
Obeticholic acid	Activator of FXR. Suppresses liver fat content by increasing Fgf19 signaling from gut to the liver.	Not tested	Yes; trials ongoing	Yes; trials ongoing	Promising results for NASH patients. Approved drug for cholangitis.	[152]
Sglt2 inhibitor	Inhibits tubular absorption of sugars in the kidney	yes	Yes; trials ongoing	Yes; trials ongoing	Bone fractures, frequent urination, urinary tract infections.	[153,154,155,156,157]
ACC + DGAT Inhibitor	Inhibits the activity of enzymes that stimulate lipogenesis in the liver	Not tested	Yes; trials ongoing	Not assessed		[158,159]
SCD1 inhibitor	Suppresses synthesis of saturated fats in the liver	Not tested	Yes, trials ongoing	Yes; trials ongoing		[160]

## Data Availability

Not applicable.
